# Supplementary material for: Patterns of the COVID-19 pandemic spread around the world: exponential versus power laws
Source: J R Soc Interface. 2020 Sep 30;17(170):20200518. doi: 10.1098/rsif.2020.0518 (PMC7536045; doi:10.1098/rsif.2020.0518)
Supplement: Additional details of the analysis [file rsif20200518supp1.pdf]

Patterns of the COVID19 pandemic spread around the world: exponential vs power laws

Natalia L. Komarova, Luis M. Schang and Dominik Wodarz

Supplementary information

1 Details of the fitting

Here we present the details of the fitting procedure used to determine the growth laws for different countries. It is illustrated with the example of Italy in figure S1. The full data for the number of cases per million in Italy are presented in figure 1(b), orange curve. In figure S1, the subset of the same data starting from 1 case per million, is plotted on the log-log scale (panel (a)) and on a linear scale (panel (b)), with varying horizontal shift, which corresponds to changing the position of time zero. This is what we refer to as a "fitting frame". The fitting frame number one is when the first data point in the selected subset corresponds to day 1. The ith fitting frame shifts this point to day i. For each fitting frame, we obtained the best fit with function $a_1 x^{b_1}$, by using a built-in Mathematica routine "FindFit". Note that the natural logarithm of the data was fitted for the figures presented here.[1]

Clearly, some fits are better than others, see figure S1(a,b). The fitting error for each fitting frame, i, was calculated as the distance between the data and the fit, normalized by the number of points:

$$\text{Err}_i = \frac{1}{N}\sqrt{\sum_{j=1}^{N}(\ln y_j^{(i)} - \ln x_j^{(i)})^2}, \tag{1}$$

where $y_j^{(i)}$ is the jth component of the dataset in frame i, $x_j^{(i)}$ is the corresponding prediction of the fit and N is the number of points in the dataset. These errors are shown for the different fitting frames in panel (c) of figure S1. The minimum error corresponds to the power $b_1 = 4.3$.

Exponential fits of the same data from Italy are shown in figure S2(a,b). In panel (a) we used a log scale, such that the exponential fits look like straight lines. It is clear that, first of all, these fits are all parallel lines and thus the error is exactly the same (thus the exponential fitting errors as functions of the fitting frame are horizontal lines, see figures 3 and 4. Second, we note that these fits are not very good for Italy, and that is why the power fit errors are always below the exponential error, see figure 4(b). For comparison, panels (c) and (d) of figure S2 show the exponential fits for the US data. We can see that the quality of the fits is better, see also figure 4(b).

[1] We also tried fitting the function without taking the logarithm; similar results were obtained (not shown).

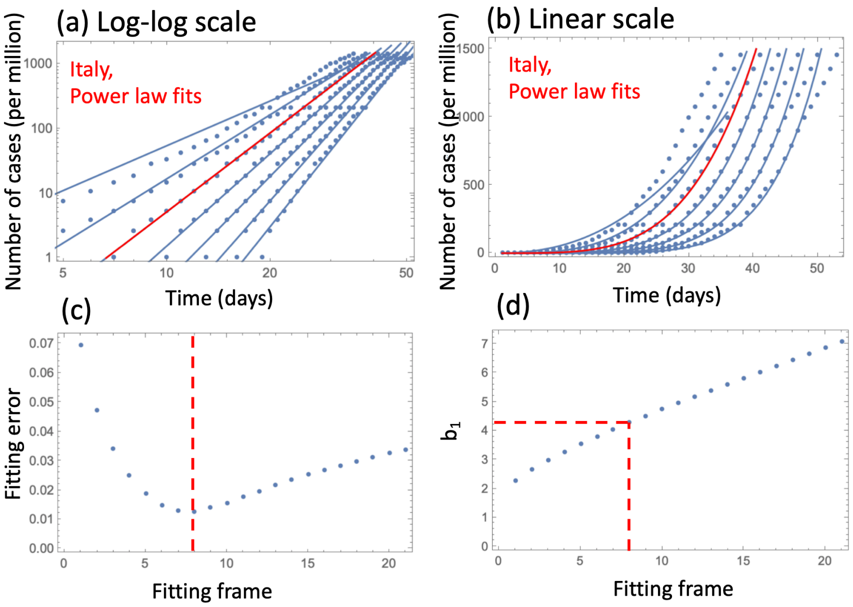

Figure S1: Details of the fitting procedure, using the example of Italy. Power law fits are presented for 7 choices of the fitting frame from 1 to 21, using (a) the log-log scale and (b) the linear scale. The data are plotted as dots and the fits as lines. The best fit is marked by the red line. (c) The power law fitting errors as a function of the frame. (d) The fitted value of the power exponent, b_1, as a function of the fit. The red dashed lines in (c) and (d) mark the best fit.

Akaike Information Criterion (AIC). Upon performing the fitting procedure as described above, we split the 61 qualifying countries into 4 groups: “Exponential”, “Exponential-like”, “Power law”, and “Power law-like”. Here we check our conclusions by applying the AIC. The AIC value is defined as $2k - 2\ln(L)$, where k is the number of parameters and L is the maximum likelihood function. The criterion selects the model with a lower AIC value as the more powerful. Here we compare two models, the exponential model that has $k = 2$ fitting parameters, and the power law model, which has $k = 3$ fitting parameters, because it contains the “frame-shift” parameter. We have for dataset i:

$$\mathrm{AIC}_i^{exp} = 2 \times 2 + N \ln \mathrm{RSS}_i^{exp} + C, \quad \mathrm{AIC}_i^{pow} = 2 \times 3 + N \ln \mathrm{RSS}_i^{pow} + C,$$

where RSS_i^{exp} is the residual sum of squares when fitting dataset i with the exponential model, RSS_i^{exp} is the same for fitting with the power law, and C is a constant that depends on the number of points in the dataset and is the same for the exponential and power law models. Further we note that the residual sum of squares is connected with the error defined in (1):

$$\mathrm{Err}_i = \frac{1}{N}\sqrt{\mathrm{RSS}_i}.$$

In order for the AIC to select the power law model as the better model for set i, we need

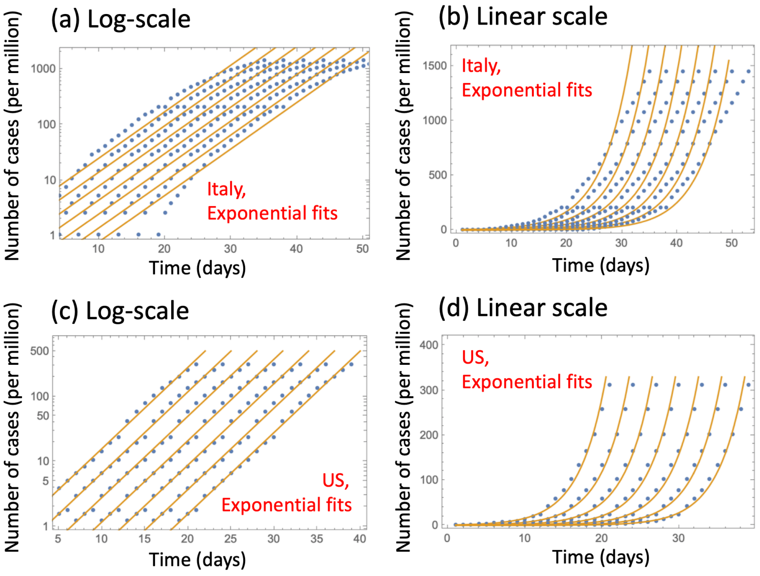

Figure S2: Examples of exponential fits for Italy (a,b) and the US (c,d), where the log scale is used in (a,c) and the linear scale in (b,d). The yellow lines show exponential fits for 7 different frames from 1 to 21, as in figure S1(a,b).

$\text{AIC}_i^{exp} - \text{AIC}_i^{pow} > 0$. This is equivalent to condition

$$\frac{\text{Err}_i^{exp}}{\text{Err}_i^{pow}} > e^{\frac{1}{N}}, \tag{2}$$

where N is the number of points in the set. If inequality (2) is reversed, then the exponential model is more powerful one according to AIC. Applying this condition to the four groups of countries that we have identified, we have obtained the following results:

- For countries that were identified "exponential", the AIC always chose exponential model as the more powerful one. This can be seen immediately from inequality (2), where in the case of "exponential" group, the left hand side is smaller than unity and the right hand side is larger than unity.

- For countries identified as "exponential-like", AIC may in some cases select the exponential and in others the power law model, but if it chooses power law, the resulting power is large (> 5), and we therefore classify such counties as exponential -like.

- For countries identified as "power law' or "power law-like", inequality (2) holds in all the cases, that is, according to AIC, a power law is always a better model in those cases. Note that since the number of points in our sets is always 15 or mode, in order for inequality (2) to be satisfied, the exponential error must be more than 7% larger than the smallest power law error ($e^{1/15} \approx 1.069$). This condition is

satisfied for all the countries in the power law and power law-like groups, see figure **??**.

Varying country selection criteria. In the main text and in this Appendix so far, we describe a fitting procedure where the "confirmed cases" data for each country were used only if the numbers exceeded 1 case per million. Here we demonstrate how this changes if a different choice is made and a minimum of 5 cases per million is required for each data point to be included. In figure S3 we demonstrate the difference for 30 countries. The choice of countries for this graphics was somewhat arbitrary: we included the 30 countries out of the subset used in figure 3 that had the largest infection (cases per million).

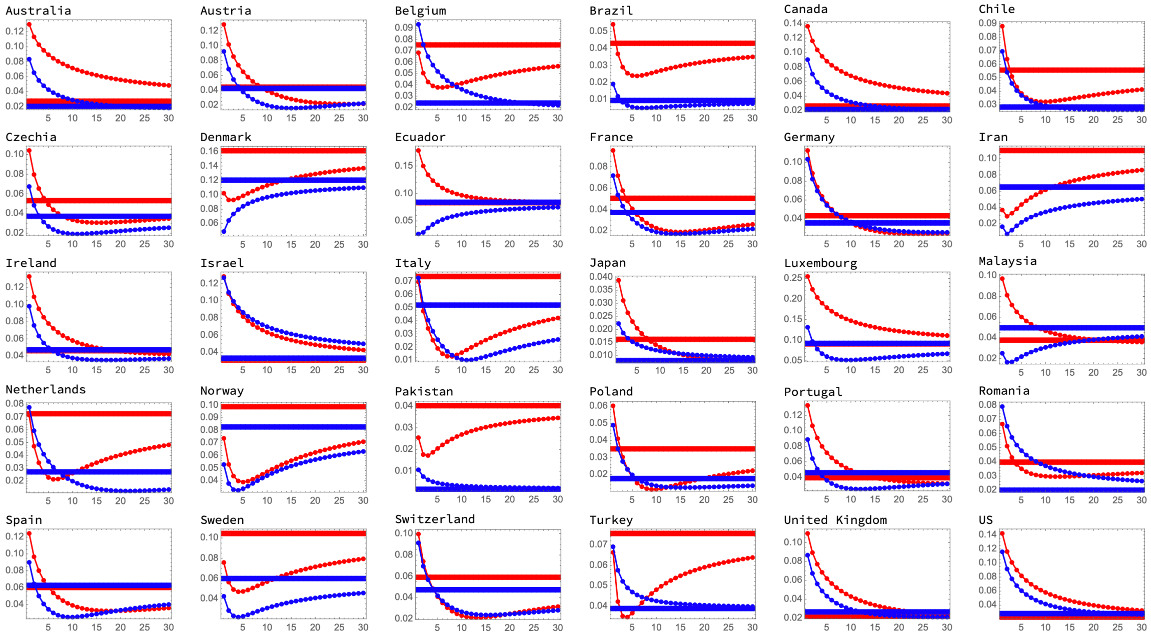

Figure S3: Comparison of two different choices of the fitting procedure, for 30 countries. Each panel represents a country, with the hrizontal axes being the fitting frame, and the vertical the fitting error. Red symbols correspond to the 1 case per million threshold, and blue circles to the 5 cases per million threshold. Circles represent the error of the power law fitting; they form non-constant functions. Squares represent the error of the exponential fitting and form horizontal lines, because these errors do not depend on the fitting frame.

Different data sources. While all the results presented here are based on the analysis of the John Hopkins data [1], we spot checked to confirm that data obtained directly from individual country databases gives similar results. Figure S4 for example plots the number of cases from the John Hopkins database (blue) and from the data found on the official Italian government website [2], yellow. There is no significant difference in the number of cases.

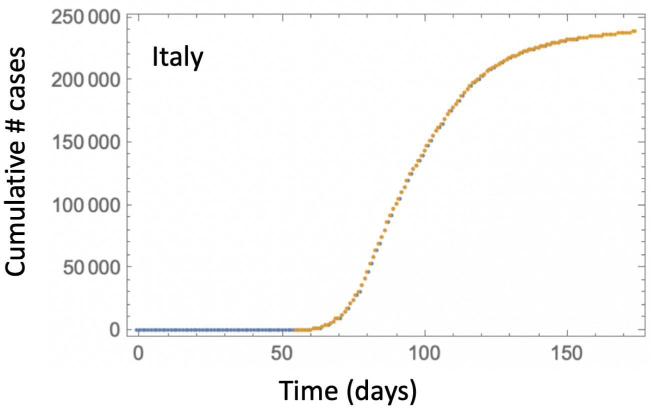

Figure S4: Comparison of the number of cases for Italy taken from the John Hopkins dataset [1], blue, and from the data found on the official Italian government website [2], yellow.

2 Best fits for different countries

Here we present plots of the best fits for different classes of countries. Figure S5 shows the 19 countries that were classified as a power law countries. The plots are presented on a log-log scale, such that the power law fits are straight lines. We can see that the best power law fit (blue) is a visibly better match than the exponential fit (yellow). Note that for all of these countries the power law fitting error for any frame shift is smaller than that obtained by the exponential fitting. The rest of the power law countries (those that were classified as power law like) are shown in figure S6. This list contains 24 countries. For convenience, we present both a log log plot (such that the power law fits, blue, appear as straight lines) and a log plot (such that the exponential fits, yellow, appear as straight lines).

Figure S7 shows the 9 countries that are characterized by a straight exponential growth. For these data, we used a log scale, such that the exponential fits are straight lines. For all of these countries the power law fitting error for any frame shift is larger than that obtained by the exponential fitting. Note however that power fits that are almost as good as exponential fits can always be found, if we we shift the frame far enough. These fits correspond to very large values of the power coefficient b_1 in the power law, see for example figure 4(a) which presents the example of the US. As the fitting frame index increases, the power law fitting error (top, blue line) approaches the exponential fitting error (horizontal yellow line). This, however, is meaningless, and does not indicate the presence of a power law. Figure S8 presents the rest of the countries from the exponential class, that is, those that were classified as exponential-like.

3 Analysis of testing data

The daily number of cases as well as the cumulative number of cases analyzed in this

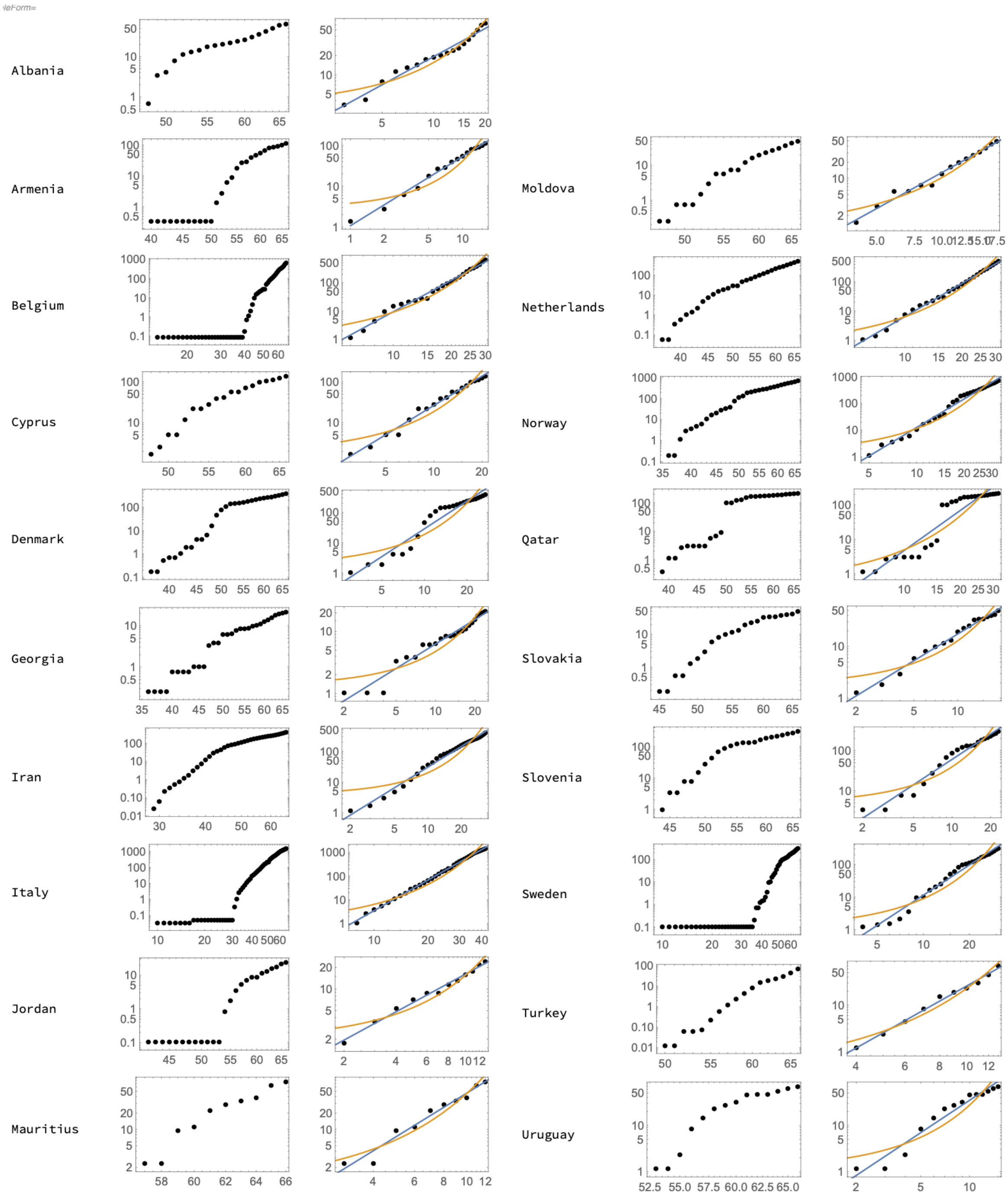

Figure S5: The 19 countries that were classified as those following a power law. For each country, two panels are presented. One is the full data (cases per million) plotted on a log log scale. The other is the subset of data (with 1 or more cases per mission) plotted on a log-log scale (black circles) together with the best power law (blue line) and exponential (yellow line) fits.

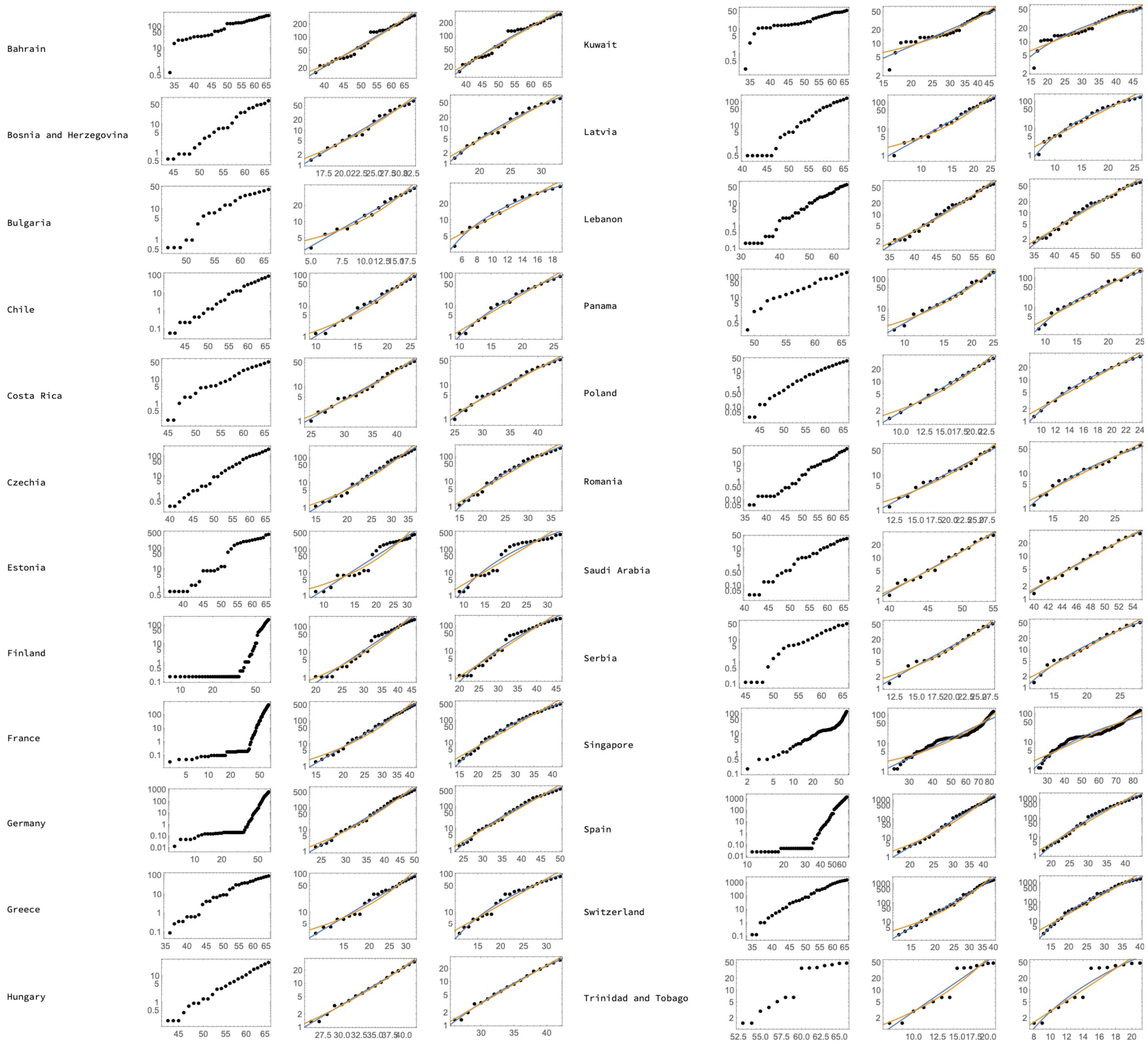

Figure S6: The 24 countries that were classified as power law like. For each country, three panels are presented: (1) is the full data (cases per million) plotted on a log log scale. (2) is the subset of data (with 1 or more cases per mission) plotted on a log-log scale (black circles) together with the best power law (blue line) and exponential (yellow line) fits. (3) is the same as (2) except on a log scale.

paper depend on the testing that is performed in individual countries. From the data on testing that are available from Our World in Data [3], we see that among the 61 countries that we classified according to the law of infection spread (Table 1), 34 contain data on testing for the time-period of interest with 5 or more data points. Among these, 25 show a statistically significant increase in the number of tests (and the other 9 do not show increase or decrease). Figure S9 presents all the testing data available for these countries and provides a linear fit of the logarithm of the test numbers together with the p-value to assess statistical significance.

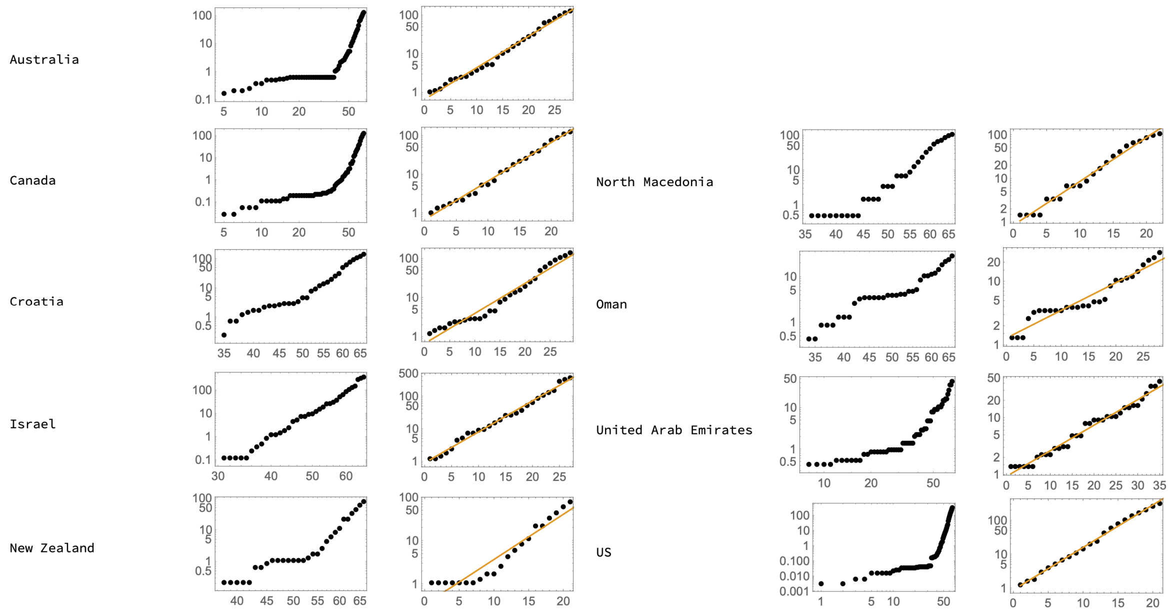

Figure S7: The 9 countries that were classified as those following an exponential law. For each country, two panels are presented. One is the full data (cases per million) plotted on a log log scale. The other is the subset of data (with 1 or more cases per mission) plotted on a log scale (black circles) together with the exponential fit (yellow line). Note that for these countries, the power law fits correspond to very high values of the exponent and are therefore not significantly different from the exponential fits.

4 Analysis of death data

COVID-related death statistics are available from Our World in Data [3]. We note that during the period covered in the analysis, only 20 out of 61 countries in table 1 report more than 20 deaths. For these countries, we fitted exponential and power law growth laws to the cumulative death time-series, and determined which fit yielded a smaller mean squares error (the same results hold if we compare R squared of the fits). Among the 20 cases, for the countries that we classified as exponential and exponential-like, 50% exhibit death time series that are better described by a power law. For the countries that we classified as power-law and power law-like, 75% exhibit death time series that are better described by a power law.

We further extended the period for the death analysis by 10 days, reasoning that there is a lag between diagnosis and death. In this extended set, the number of countries with more than 20 deaths almost doubles (38 countries out of the 61 for which we provide classification). For the countries that we classified as exponential and exponential-like, 75% exhibit death time series that are better described by a power law. For the countries that we classified as power-law and power law-like, 85% exhibit death time series that are better described by a power law. Figure S10 shows examples of fits for 6 cases, where in 3 of them exponential fits are better and in the other 3 cases power-law fits are better.

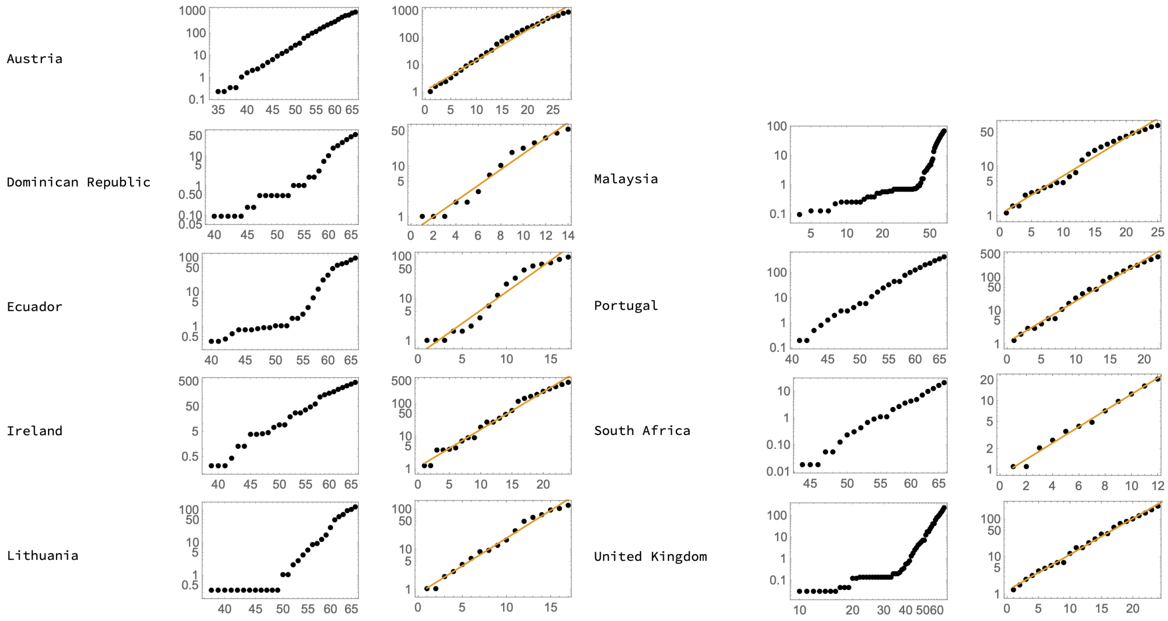

Figure S8: The 9 countries that were classified as exponential-like. Panels are as in figure S7.

5 Additional analysis

In this paper we show that while many countries exhibit initial exponential-like growth, it subsequently slows down to a power-like growth. Here (see figure S11) we show examples of simulations where we explore the scenario of continual exponential growth, spanning the whole time-frame of the dataset. One can see that there are orders of magnitude difference in the case number under exponential growth, compared to the actual case count.

References

[1] Novel Coronavirus 2019; 2020. `https://datahub.io/core/covid-19#data-cli`.

[2] Dipartimento della Protezione Civile COVID-19 Italia - Monitoraggio della situazione; 2020. `http://opendatadpc.maps.arcgis.com/apps/opsdashboard/index.html#/b0c68bce2cce478eaac82fe38d4138b1`.

[3] Our World in Data; 2020. `https://ourworldindata.org/grapher/full-list-total-tests-for-covid-19`.

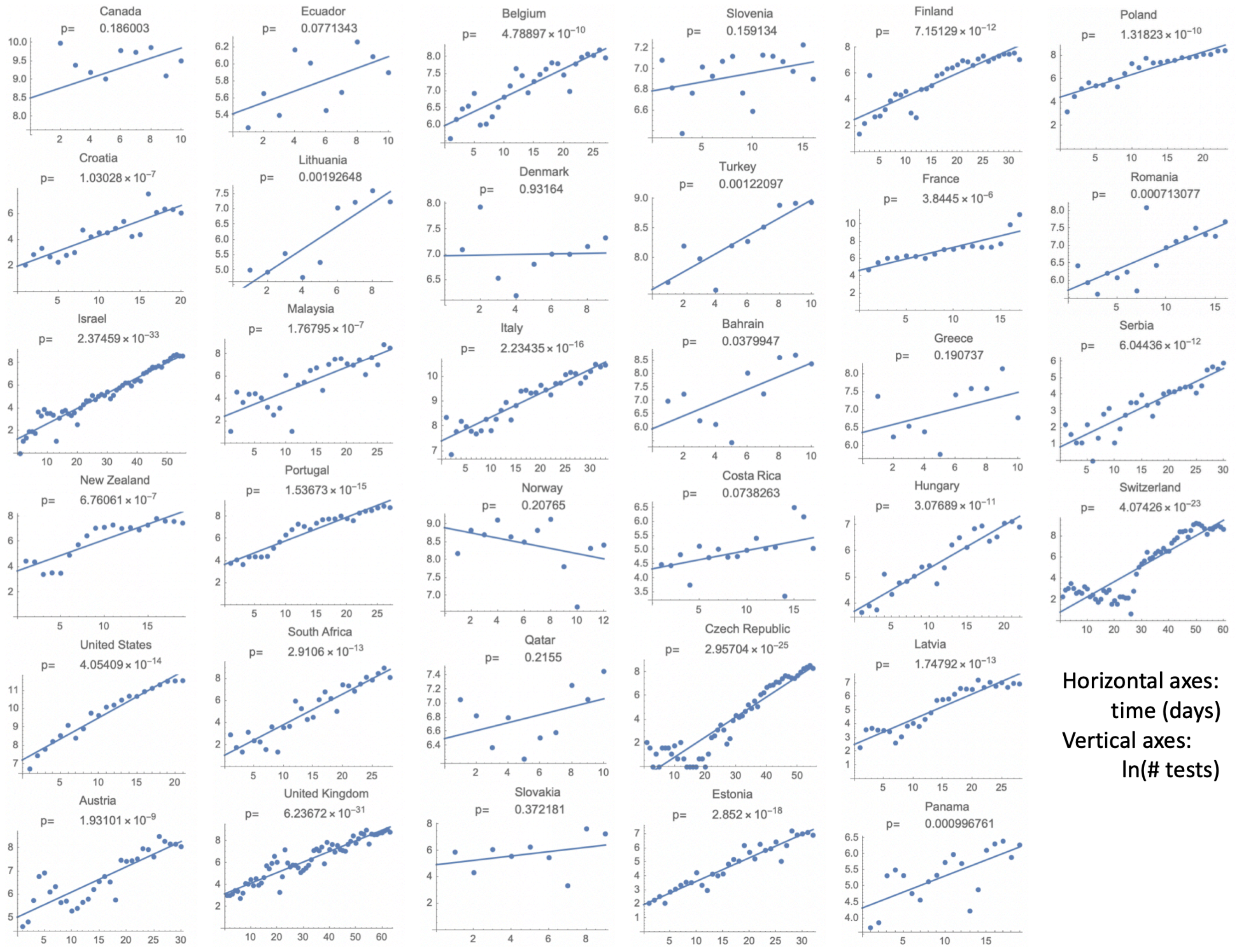

Figure S9: The (natural log) number of daily tests for countries in Table 1, where the last temporal point corresponds to March 28, 2020. The best linear fit of the log data is presented in every panel as a straight line.

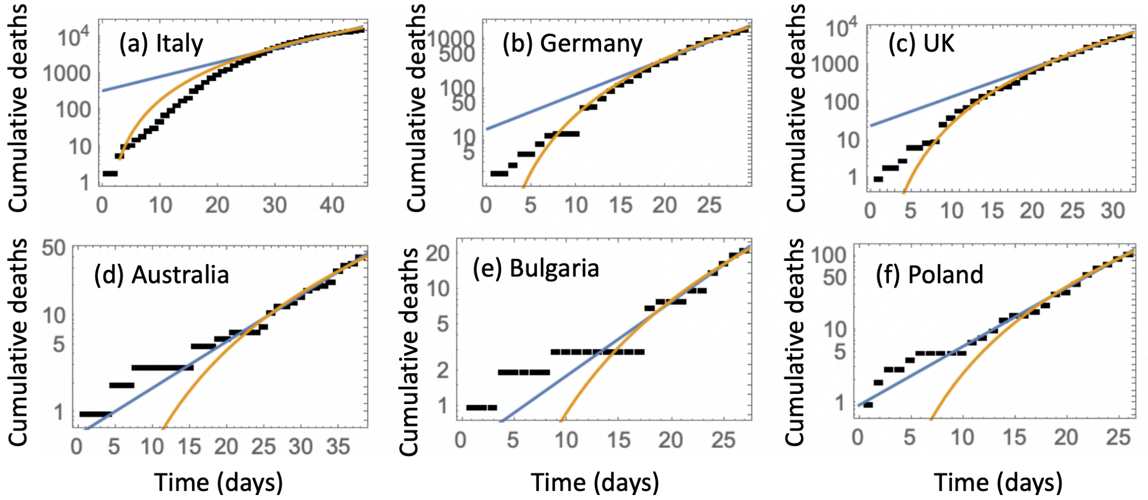

Figure S10: Examples of 6 countries showing the cumulative death data (black) together with the best exponential (blue) and power law (orange) fits. (a-c) The power-law fit results in a smaller error. (d-e) Exponential fit resultsv in a smaller error.

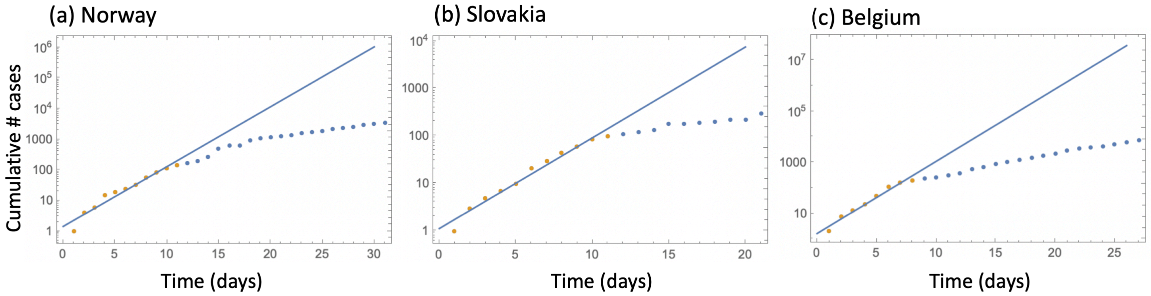

Figure S11: Three examples of countries that were classified as "power law", where a simulation was performed to compare the actual infection growth (blue points) with a growth that continues the initial exponential-like expansion (found as the best exponential fit of the first 10 points marked by yellow) and shown as straight lines. The first data point corresponds to the first point with 2 cases, and the last data point is March 28, 2020.
